# Motor cortex repetitive transcranial magnetic stimulation in fibromyalgia: a multicentre randomised controlled trial

**DOI:** 10.1016/j.bja.2024.12.045

**Published:** 2025-03-13

**Authors:** Valquíria A. Silva, Abrahão F. Baptista, Alessandra S. Fonseca, Adriana M. Carneiro, André R. Brunoni, Paulo.E.M. Carrilho, Catarina C. Lins, Gabriel T. Kubota, Ana Mércia B.L. Fernandes, Jorge.D.S. Lapa, Lucas M. dos Santos, Ivo Sasso, Katia Monte-Silva, Frédérique Poindessous-Jazat, Nobuhiko Mori, Kenji Miki, Adriana Baltar, Clarice Tanaka, Manoel J. Teixeira, Koichi Hosomi, Didier Bouhassira, Nadine Attal, Daniel Ciampi de Andrade

**Affiliations:** 1 LIM 62 - Pain Center, Department of Neurology, São Paulo, Brazil; 2 Service of Interdisciplinary Neuromodulation (SIN), Department and Institute of Psychiatry, São Paulo, Brazil; 3 Medical-Surgical Nursing Department, University of São Paulo, São Paulo, Brazil; 4 Center for Mathematics, Computation and Cognition, Federal University of ABC, São Paulo, Brazil; 5 Mood Disorder Program (Pro-GRUDA), Department of Psychiatry, University of São Paulo, São Paulo, Brazil; 6 Medicine School “Professor Orival Alves”, State University of Western Paraná (UNIOESTE), Paraná, Brazil; 7 Neurosurgery Unit, Hospital de Cirurgia, Aracaju, Sergipe, Brazil; 8 Department of Medicine, Federal University of Sergipe, São Cristóvão, Sergipe, Brazil; 9 LIM 54 - Physiotherapy Research Laboratory, University of São Paulo, São Paulo, Brazil; 10 Applied Neuroscience Laboratory, Universidade Federal de Pernambuco, Cidade Universitária, Pernambuco, Brazil; 11 U987, UVSQ-Paris-Saclay University, Ambroise Paré Hospital, Boulogne-Billancourt, France; 12 Department of Neurosurgery, Osaka University Graduate School of Medicine, Osaka, Japan; 13 Faculty of Health Science, Osaka Yukioka College of Health Science, Osaka, Japan; 14 Center for Pain Management, Hayaishi Hospital, Osaka, Japan; 15 Center for Neuroplasticity and Pain (CNAP), Department of Health Science and Technology, University of Aalborg, Gistrup, Denmark

**Keywords:** chronic pain, clinical trial, fibromyalgia, neuromodulation, transcranial magnetic stimulation

## Abstract

**Background:**

Despite affecting 2–4% of the population worldwide, fibromyalgia often remains refractory to treatment. Here we report the first international randomised double-blind, sham-controlled trial developed to assess the efficacy of repetitive transcranial magnetic stimulation (rTMS) as an add-on therapy for fibromyalgia.

**Methods:**

Women aged ≥18 yr with fibromyalgia refractory to best available treatment were enrolled in Brazil, France, and Japan, and randomised to 10 Hz motor cortex (M1) rTMS, 3000 pulses day^−1^, or sham stimulation. This included 10 induction sessions over 2 weeks, followed by weekly maintenance (6 weeks), and fortnightly extended maintenance (8 weeks). Primary outcome was ≥50% pain reduction at week 8 compared with baseline. Secondary outcomes included pain interference, mood, global impression of change, and Fibromyalgia Impact Questionnaire (FIQ) scores at weeks 8 and 16.

**Results:**

We randomised 101 women (mean age 48 [range 25-83] yr) into active (*n*=52) or sham (*n*=49) arms. Bayesian analysis revealed a 99.4% probability of ≥50% pain reduction at week 8 in the active group *vs* sham (odds ratio [OR] 3.04; 95% credible interval [95% CrI] 1.26–8.06), with a number needed to treat of 4.54. Frequentist analysis confirmed that relative pain reduction was higher in the active than in the sham group (40.4% *vs* 18.4%, *P*=0.028). At week 16, this probability reduced to 34.2% (OR 0.815; 95% CrI 0.313–2.1), but the likelihood of FIQ score reduction was 79.1%. The intervention appeared safe.

**Conclusions:**

Add-on M1-repetitive transcranial magnetic stimulation reduced pain intensity up to 8 weeks in women with fibromyalgia. Although analgesic effects waned, functional improvements remained during extended maintenance at week 16.

**Editor's key points**

• Fibromyalgia affects 2–4% of the population worldwide, and often remains refractory to treatment.
• In this international, randomised, double-blind, sham-controlled trial, repetitive transcranial magnetic stimulation was effective as an add-on therapy for fibromyalgia.
• Repetitive transcranial magnetic stimulation produced pain relief in women with fibromyalgia up to 8 weeks after treatment, with an excellent effect size and apparently good safety profile.
• Future efforts are needed to characterise the response profile further and personalise therapy to improve the response rate further.

Fibromyalgia is a chronic and complex pain disorder, affecting 2–4% of the population worldwide, especially women.^1^ Despite reporting widespread pain referred to the musculoskeletal system, people with fibromyalgia do not have abnormalities in muscles, joints, or other somatic tissues. When present, peripheral changes in fibromyalgia occur only in a proportion of patients and do not correlate with symptoms.^1^^,^^2^ Instead, fibromyalgia is associated with central abnormalities leading to gain in sensory processing.^3^ Clinical correlates of central sensitisation have been reported in fibromyalgia, and are considered to correlate with symptom intensity.^4^

Fibromyalgia has been associated with low motor cortex GABA-dependent cortical inhibition, with decreased tone of descending pain modulatory mechanisms.^3^ Fibromyalgia has also been associated with decreased connectivity between nociceptive integration areas such as the insula, broader neuronal networks such as the default mode network,^5^ and areas highly connected to specific segments within the primary motor cortex, which are not directly involved in distal limb motor control.^6^

Fibromyalgia is treated with pharmacological and nonpharmacological approaches. Medications approved for fibromyalgia have low effect sizes when used as monotherapy.^7^ Conversely, nonpharmacological therapies such as noninvasive neuromodulation have broader effects on nonpainful symptoms such as mood and fatigue.^7^ Irrespective of the treatment strategy, a substantial proportion of patients currently remain symptomatic despite the best medical treatment available.

Among the nonpharmacological approaches for chronic pain is repetitive transcranial magnetic stimulation (rTMS). Primary motor cortex (M1) stimulation with rTMS has been explored in other chronic pain conditions, mainly neuropathic pain, and is currently indicated for treating the latter.^8^^,^^9^ Beyond voluntary motor control, M1 has areas that are highly connected to extra-motor networks responsible for interoceptive, cognitive, and nociceptive control.^6^ Its analgesic effects in experimental pain models and patients depend on the release of endogenous opioids^10^ and the availability of NMDA-type glutamate receptors.^11^ Analgesic effects of M1-rTMS have been shown to influence phase synchronisation between motor and prefrontal areas for several minutes after the end of the stimulation session,^12^ and to be maintained up to months with spaced maintenance sessions.^13^

In smaller trials, rTMS has been tested in people with fibromyalgia, with stimulation targeting mainly M1^14^, ^15^, ^16^ or the dorsolateral prefrontal cortex.^17^, ^18^, ^19^ Results of these trials are conflicting owing to several factors, such as the nonsystematic inclusion of fibromyalgia patients with comorbid major depression,^18^^,^^19^ heterogeneous outcome measures,^14^ relatively small sample sizes,^14^^,^^15^ and short follow-up.^17^^,^^18^ More importantly, key stimulation parameters were frequently either not reported^14^^,^^15^ or were suboptimal.^16^ Building upon these previous studies and taking into consideration the stimulation parameters with the best consistent responses, we performed the first international multicentre study of the effects of M1-rTMS in a large international sample of people with fibromyalgia.

## Methods

This international, multicentre, double-blind, parallel-group, sham-controlled randomised trial was conducted in pain and neurology centres in Brazil (Federal University of Pernambuco, State University of Western Paraná, and two centres in the University of São Paulo: the Neurology and Physiotherapy Departments), France (Centre d’Evaluation et de Traitement de la Douleur Hôpital Ambroise Paré, Paris-Saclay University), and Japan (Department of Neurosurgery, Osaka University).

The trial was approved by all respective institutional review boards (coordination centre # 96090518.0.1001.0068), and registered at clinicaltrials.gov (NCT03658694) on September 2018 before the first participant was enrolled. The Good Clinical Practice and Clinical Research Act of Japan guidelines were followed, and the study complied to the Declaration of Helsinki. All participants or their representatives provided written informed consent before enrolment.

### Data safety

Randomisation, data collection, and data storage were performed using REDCap software (Nashville, TN, USA), and data monitoring was led by a team of researchers not involved in data collection or rTMS delivery.

### Participants

We included women aged ≥18 yr, diagnosed with chronic pain caused by fibromyalgia according to the 2016 revision of the American College of Rheumatology criteria.^20^ According to these criteria, the following conditions are needed for diagnosis: (1) widespread pain index (WPI) ≥7 and symptom severity scale (SSS) score ≥5; or WPI of 4–6 and SSS score ≥9; (2) generalised pain, defined as pain in four or more out of five body regions; and (3) symptoms present for at least 3 months. Participants were only included if they were able to understand the study protocol. Subjects were excluded for known alcohol or illicit drugs use disorder, known major psychiatric conditions, contraindications to rTMS (i.e. recent head trauma or concussion, metal implants in the skull, pregnancy, cardiac pacemaker), no contraceptive method if in childbearing age (i.e. intrauterine device or oral contraceptive), medical conditions that require hospitalisation, or currently participating in other clinical studies.

Participants were prospectively recruited from outpatient clinics in the hospitals and associated clinics from the study centres, or by their respective primary caregivers who had no other role in the study.

### Interventions

Participants were randomised at 1:1 ratio into active and sham rTMS treatments, which both consisted of induction sessions (daily for 10 days), followed by maintenance (weekly for 6 weeks), and extended maintenance (fortnightly for 8 weeks). rTMS was delivered as an add-on treatment to each participant's steady therapeutic regimen. During the study, participants were instructed to maintain their baseline fibromyalgia treatments unchanged, if possible. During sessions, all participants sat in a comfortable reclining chair and were instructed to remain as relaxed as possible.

Each rTMS session consisted of 30 trains of 100 pulses delivered at 10 Hz to the motor hot spot of the hand representation on the left M1 with a 20-s intertrain interval, totalling 3000 pulses per session for a total duration of 15 min. The stimulation intensity was set at 80% of the resting motor threshold, which was defined as the minimal stimulation intensity required for evoking a 50 μV of electromyographic response of the right first dorsal interosseus muscle in at least five out of 10 measurements. Active rTMS stimulation was performed with active figure-of-eight coils (MagVenture Tonika Elektronic, Farum, Denmark), with the handle oriented backwards and aligned 45 degrees from the sagittal plane. Sham stimulation was conducted with respective identical sham coils (MagVenture Tonika Elektronic) of identical size, colour, and shape, emitting a sound resembling that emitted by the active coil. Details of the coil models used in each of the centres and the use of neuro-navigation and of robots during sessions are provided in the Supplementary Table S1.

### Randomisation and masking

A random sequence was generated using the randomisation module in REDCap^21^ in blocks of six per centre. All data were collected and stored using REDCap, with password-protected privileges according to each researcher's role in the study (data collection, randomisation, and stimulation system manipulation). The randomisation schedule was kept confidential to the researchers, and allocation concealment was achieved by dedicated access restriction to the REDCap platform. All researchers participating in this study were blinded to randomisation, except for the rTMS operators who were aware of the type of rTMS being applied (active or sham), but were blinded to all other assessments and had no other role in the study. Care was taken not to set participant appointments simultaneously so that waiting-room interactions could be avoided and blinding integrity preserved.

### Procedures

Recruitment was carried out from November 2019 to February 2022, with the last follow-up period ending in October 2022. Participants were thoroughly assessed at baseline, encompassing detailed personal, clinical, and sociodemographic data. Data on main and secondary outcomes, including the incidence of side-effects were collected at weeks 8 and 16. Mean pain intensity during the previous 24 h before the stimulation session was scored before each rTMS application.

### Blinding assessment

Blinding assessment was performed at week 16, after the last stimulation session at the end of the study (Supplementary Fig. S1). According to a published protocol,^22^ this procedure involved asking all participants: (1) if they were able to tell which sequence of treatment they were allocated to (yes or no); (2) which sequence they think they received (active or sham); and (3) if they would like to maintain the treatment sessions for a longer period, should this option be offered to them (yes or no). The answers to these questions were compared between sham and rTMS groups.

### Outcomes

The primary outcome was the proportion of participants reporting a reduction ≥50% of the mean pain intensity at week 8, compared with baseline. Mean pain intensity reflected the average in the past week and was measured with a verbal numerical rating scale (NRS),^23^ an 11-point scale that is anchored at 0 (no pain) and 10 (maximal pain imaginable). Secondary outcomes included a direct comparison of results from subjects allocated to the active and sham arms at 8 and 16 weeks and included average pain intensity and interference scores from the Brief Pain Inventory (BPI)^23^; mean scores from the Fibromyalgia Impact Questionnaire (FIQ),^24^ Hospital Anxiety and Depression Scale (HADS),^23^ and Global Impression of Change questionnaire (GIC)^23^; and on-going medication use for fibromyalgia treatment. The estimated differences, odds ratios (ORs), and hypothesis probabilities were calculated. Pain intensity within the previous 24-h presence of side-effects were obtained after each rTMS session. A responder prediction analysis was performed to assess baseline characteristics that could predict the main outcome at week 8 in the active arm.

### Safety assessment

After each treatment session, adverse events were assessed using a structured questionnaire, based on the most frequently reported rTMS side-effects, in which participants were actively asked about the occurrence of adverse events and their relationship with the stimulation. The severity of adverse events was classified as mild, moderate, or severe. Severe adverse events were defined as those requiring close medical monitoring, intervention, or hospitalisation, or which resulted in serious health problems, prolonged disability, or death.

### Statistical analyses

Before the trial began, various assumptions were explored, including minimal intervention differences, standard deviations, and dropout rates, leading to the decision to include 50 participants per arm to account for potential dropouts. The study was suspended during the COVID-19 pandemic, and 32 patients were excluded from the data analysis after a steering board decision, with recruitment resuming post-pandemic. Analyses were conducted using an intention-to-treat (ITT) approach; exploratory analyses involved visual evaluations and standardised mean differences to assess effect sizes. A Bayesian multilevel model analysed the impact of rTMS on pain outcomes, incorporating multivariate imputation for missing data and using Markov Chain Monte Carlo method for statistical sampling. Additionally, frequentist methods were used for baseline (anova and Student's *t*-test) data and primary outcome (χ^2^ test) analysis. All analyses were performed with R statistical software.^25^ Details about statistical analyses can be found in the Methods section of Supplementary material.

## Results

### Baseline characteristics and study flowchart

**Fig 1:**
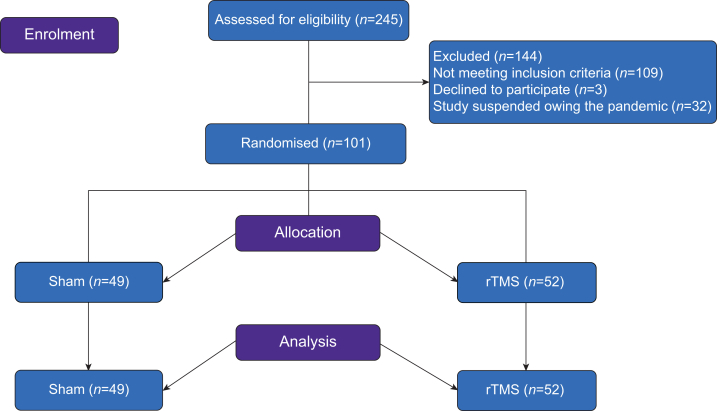
Study flowchart. rTMS, repetitive transcranial magnetic stimulation.

Enrolment occurred from November 2019 to February 2022. Out of 245 participants screened, 101 were included and randomised. Most of these participants were enrolled at research sites in Brazil (*n*=79), followed by those in France (*n*=12) and Japan (*n*=10) (Supplementary Table S2). The main reasons for exclusion were related to the eligibility criteria (*n*=109), not consenting (*n*=3), and study suspended because of the COVID-19 pandemic (*n*=32) (Fig 1). The ITT analysis included 52 participants from the rTMS arm and 49 from the sham arm. Baseline participant characteristics are shown in Table 1. No differences across centres were found for the main baseline demographic features, primary outcomes, and secondary outcomes (Supplementary Table S2).

**Table 1:** Baseline pain and participant characteristics. Data are presented as mean (sd) or *n* (%); unless otherwise stated, *P*-values refer to the comparison between rTMS and sham treatments. BPI, Brief Pain Inventory; FIQ, Fibromyalgia Impact Questionnaire; HADS, Hospital Anxiety and Depression Scale; NRS, verbal numerical rating scale; rTMS, repetitive transcranial magnetic stimulation.

	Total (*n*=101)	rTMS (*n*=52)	Sham (*n*=49)
**Age (yr), mean (range)**	48.1 (25–83)	49.0 (25–79)	47.1 (31–83)
**Race, *n* (%)**			
White	52 (51.5)	25 (48.1)	27 (55.1)
Black	22 (21.8)	12 (23.1)	10 (20.4)
Asian	10 (9.9)	5 (9.6)	5 (10.2)
Other	17 (16.8)	10 (19.2)	7 (14.3)
**Education, *n* (%)**			
Elementary	15 (14.9)	7 (13.5)	8 (16.3)
Middle	19 (18.8)	11 (21.2)	8 (16.3)
High	26 (25.7)	14 (26.9)	12 (24.5)
Undergraduate or more	41 (40.6)	20 (38.5)	21 (42.9)
Average pain intensity NRS	7.82 (1.23)	7.9 (1.27)	7.73 (1.19)
BPI worst pain intensity in the past 24 h	8.81 (1.19)	8.67 (1.34)	8.96 (0.999)
BPI Pain Interference Score	7.96 (2.12)	7.92 (2.05)	8 (2.21)
HADS Depression	8.98 (4.25)	8.63 (4.47)	9.35 (4.02)
FIQ	68.5 (12.8)	69 (13.3)	68.1 (12.4)

### Effects on the primary outcome

**Fig 2:**
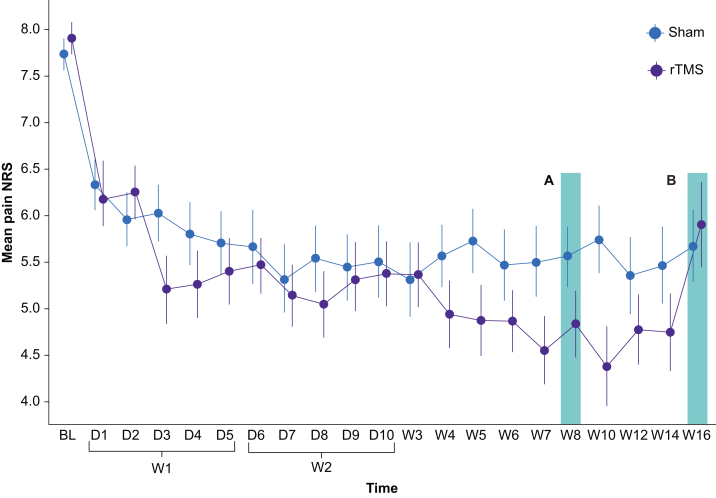
Average pain intensity. Weekly average pain intensity (0–10 point numerical rating scale [NRS]) for both the active (black line) and sham (grey line) groups. Error bars indicate standard deviation. The primary endpoint was evaluated at the 8-week follow-up (A), and the last follow-up assessment occurred at week 16 (B). BL, baseline; D, day; rTMS, repetitive transcranial magnetic stimulation; W, week.

The preplanned single-level Bayesian models demonstrated a 99.4% probability of a higher proportion of participants achieving ≥50% reduction in pain intensity by week 8 in the rTMS group compared with the sham group (estimated difference: 1.11; 95% credible interval [95% CrI] 0.232–2.09), OR of 3.04 (95% CrI 1.26–8.06). Confirmatory frequentist analysis revealed a difference between responders in the rTMS and sham groups (active, 40.4%; sham, 18.4%; *P*=0⸱028; Fig 2). The number needed to treat was 4.54, with an effect size of 0.49. Notably, no significant differences were found in the average pain intensity levels achieved at week 8 across research sites (Supplementary Table S3).

### Effects on secondary outcomes

At week 8, single-level Bayesian models demonstrated a 92.6% probability of achieving a reduction in pain intensity (estimated difference: –0.529 [–1.21–0.192]), and a 65.8% probability of reduction of pain interference in daily activities (estimated difference: –0.17; [–1.02–0.693]; Table 2), with active rTMS compared with sham stimulation. FIQ scores had a probability of improvement of 80.1% (estimated difference: –2.95 [–9.72–3.83]) when compared with the sham group. Anxiety levels assessment (HADS-anxiety) showed a probability of improvement after active rTMS of 67.7% (estimated difference: –0.392 [–2.14–1.3]), whereas depression scores (HADS-depression) presented a 32.1% probability of reduction (estimated difference: 0.407 [–1.28–2.12]). At this time point, when compared with sham stimulation, the probability of participant's global impression of change to be ‘much’ and ‘moderately improved’ after active rTMS was 96.8% (estimated difference: 0.759 [–0.041–1.57]), whereas the same probability for the clinician's assessment was 88.8% (estimated difference: 0.548 [–0.301–1.45]). Compared with sham treatment, active stimulation led to probabilities of reduction in the use of antidepressants and gabapentinoids of 77.3% (estimated difference: –0.434 [–1.55–0.687]) and 45.6% (estimated difference: 0.074 [–1.19–1.35]), respectively (Table 2). Similarly to the primary outcome, there were no significant differences in any of the evaluated secondary endpoints across research sites (Supplementary Table S3).

**Table 2:** Pain, functionality, and global impression of change outcomes and ongoing medication reduction after active motor cortex repetitive transcranial magnetic stimulation compared with sham treatment at the 8- and 16-week follow-ups. Data are presented as estimated difference (95% credible interval). BPI, Brief Pain Inventory; FIQ, Fibromyalgia Impact Questionnaire; GIC, Global Impression of Change; HADS, Hospital Anxiety and Depression Scale; NRS, verbal numerical rating scale.

Outcomes	Estimated difference between active and sham treatments	Probability of achieving the outcome (%)
**Week 8**		
≥50% reduction in average pain intensity NRS	1.11 (0.232–2.09)	99.4
Improvement in BPI pain severity score	–0.529 (–1.21 to 0.192)	92.6
Improvement in BPI pain relief with current medications	0.467 (–8.07 to 8.5)	45.9
Improvement in BPI pain interference scores		
Overall interference score	0.17 (–1.02 to 0.693)	65.8
Overall activities	0.439 (–1.43 to 0.589)	79.9
Mood	0.326 (–0.724 to 1.41)	26.7
Walking	0.045 (–0.992 to 1.08)	46.6
Work	–0.578 (–1.58 to 0.43)	86.9
Relationship with other people	–0.165 (–1.24 to 0.933)	61.7
Sleep	–0.846 (–2.05 to 0.339)	92.3
Ability to enjoy life	–0.352 (–1.4 to 0.722)	74.3
Improvement in FIQ scores	–2.95 (–9.72 to 3.83)	80.1
Improvement in HADS anxiety scores	–0.255 (–1.93 to 1.43)	61.8
Improvement in HADS depression scores	0.421 (–1.2 to 1.99)	29.7
Improvement in patient GIC category	0.759 (–0.041 to 1.57)	96.8
Improvement in clinician GIC category	0.548 (–0.301 to 1.45)	88.8
Reduction in medication compared with baseline:		
Antidepressants	–0.434 (–1.55 to 0.687)	77.3
Gabapentinoid	0.074 (–1.19 to 1.35)	45.6
Opioids	0.306 (–1.11 to 1.71)	33.1
Metamizole	0.199 (–1.01 to 1.44)	37.2
Other anticonvulsants	0.862 (–0.958 to 2.92)	17.5
**Week 16**		
≥50% reduction in average pain intensity NRS	–0.204 (–1.16 to 0.742)	34.2
Improvement in FIQ score	–2.45 (–8.7 to 3.53)	79.1
Improvement of clinician GIC category	0.066 (–0.794 to 0.954)	55.8
Improvement of patient GIC category	–0.069 (–0.887 to 0.709)	43.5

By week 16, the probability of the active arm having more responders (≥50% pain reduction) than the sham was 34.2% (estimated difference: 0.204 [–1.16–0.742], OR 0.815 [0.313–2.1]), which was not significant in a frequentist analysis (active rTMS responders, 19.2%; sham rTMS responders, 22.4%; *P*=0⸱878); while the probability of greater reduction of the FIQ scores in the active rTMS arm was 79.1% (estimated difference: –2.45 [–8.7–3.53]). No significant differences were found across trial centres for the main primary and secondary outcomes (Supplementary Table S3). Correlations between response to active rTMS and baseline characteristics such as age, race, educational level, and treatment revealed that participants receiving opioids were less often responders than those not taking these drugs (Supplementary Table S4).

### Adverse events

Twenty-two (21.8%) and 24 (23.8%) participants reported adverse events in week 8 and 16, respectively. There was no statistically significant difference in the incidence of such events between the sham and rTMS groups (*P*=0.691 at week 8; *P*=0.689 at week 16). The most commonly reported adverse events were headache (rTMS, 15.4%; sham, 16,3%; *p*=0.897), neck pain (rTMS, 13.5%; sham, 10.2%; *p*=0.613), and somnolence (rTMS, 13.5%; sham, 10.2%; *p*=0.613), and no individual adverse event was significantly more frequent between groups. Regarding severity, at week 8, 10.9% of patients experienced mild events and another 10.9% experienced moderate events. By week 16, these rates were 13.9% for mild events and 9.9% for moderate events. Notably, no participants in either group reported severe adverse events at any time point. Breaking down the data by group, 80.8% of the rTMS group and 75.5% of the sham group reported no adverse events at week 8. Importantly, no deaths or seizures occurred during the trial (Table 3).

**Table 3:** Participants reporting adverse events after at least one treatment session. Data are presented as *n* (%). rTMS, repetitive transcranial magnetic stimulation.

	Total (*n*=101)	Sham (*n*=49)	rTMS (*n*=52)	*P*
**Week 8**				
Any adverse event	22 (21.8)	12 (24.5)	10 (19.2)	0.69
Adverse event severity				0.57
None	79 (78.2)	37 (75.5)	42 (80.8)	
Mild	11 (10.9)	5 (10.2)	6 (11.5)	
Moderate	11 (10.9)	7 (14.3)	4 (7.7)	
Severe	0 (0)	0 (0)	0 (0)	
**Week 16**				
Adverse events	24 (23.8)	13 (26.5)	11 (21.2)	0.69
Adverse event severity				0.73
None	77 (76.2)	36 (73.5)	41 (78.8)	
Mild	14 (13.9)	7 (14.3)	7 (13.5)	
Moderate	10 (9.9)	6 (12.2)	4 (7.7)	
Severe	0 (0)	0 (0)	0 (0)	

### Blinding assessment

There was no statistically significant difference between the sham and rTMS groups regarding participants' ability to correctly guess their treatment allocation at the end of the study (Supplementary Table S5).

## Discussion

Data from this multicentre international trial show that the addition of daily, followed by weekly M1-rTMS sessions to the treatment of women with fibromyalgia provided a higher probability of reducing pain and other fibromyalgia-related symptoms, and led to positive impression of improvement up to 8 weeks. Further extension to fortnightly maintenance sessions resulted in a reduction in the probability of pain relief, despite the overall persistence of treatment effects on fibromyalgia symptoms and impression of improvement. The probability of response to real rTMS was negatively related to baseline use of opioids, but not to other baseline characteristics, or to the use of other drugs. These findings support that M1-rTMS is efficacious as an add-on therapy for people with fibromyalgia who remain symptomatic despite optimised best medical therapy.

In line with the findings of smaller trials,^13 14^ M1-rTMS did not result in improvement of depressive symptoms. Despite being advised to keep their pre-trial treatment, participants in the active arm had a greater probability of reducing doses of antidepressants along the study. This is relevant, as tricyclic antidepressants and serotonin–norepinephrine reuptake inhibitors are among the drugs with higher efficacy to control fibromyalgia pain symptoms.^26^ We hypothesise that antidepressant dose reduction reflected a subjective perception of symptom relief by patients or their primary care physicians, leading to a reduction in dose. It remains to be determined whether such voluntary reduction in antidepressants dosage influenced the negative findings on M1-rTMS effects on mood during the trial.

The analgesic effects of M1-rTMS during spaced maintenance sessions performed 14 days apart from each other were less pronounced and not significant. This contrasts to previous studies with smaller samples of people with fibromyalgia, which suggested that biweekly and monthly stimulation sessions could maintain the effects of daily induction and weekly maintenance sessions.^13 14^ Although the probability of pain relief was reduced during the extended maintenance period, the probability of reduction of fibromyalgia-related symptoms remained relatively stable. That, added to the lower use of antidepressants in the active group, supports an interpretation that responders to active rTMS were functionally less impacted by fibromyalgia symptoms despite their pain relapse.

Some baseline medications used by participants could directly interfere with the mechanisms behind analgesic M1 stimulation. The use of opioids at study entry was negatively associated with the response to M1-rTMS in our study. Several experimental^10^ and clinical chronic pain studies^27^ have suggested that the analgesic effects of M1 stimulation depend on the availability of endogenous opioids, and that opioid antagonists can abolish analgesia caused by M1 neuromodulatory approaches. Despite positive trials in the past, opioids are currently known to be unhelpful or even harmful if prescribed for long-term use and without restrictions for people with fibromyalgia. However, weak opioids are still recommended in guidelines for fibromyalgia^26^ under instances of refractory severe pain.

Blinding is a frequent challenge in therapeutic clinical trials, especially those addressing nonpharmacological treatments. In case of M1-rTMS, this is less problematic in parallel design trials, such as ours, compared with crossover studies, as all participants were naive to rTMS.^28^ In this trial, a systematic preplanned blinding assessment suggested it was successful. Another issue is allocation concealment and blinding of rTMS operators. We took care to segregate researchers performing rTMS from any other type of data collection roles, and they had only privilege to access participants' online clinical research forms concerning the rTMS session, not responses to questionnaires and other outcomes.

It has been proposed that the optimal scenario to perform sham in rTMS trials is to use automated systems where each participant has a card with a hidden code indicating their arm allocation in the study.^29^ Such systems allow researchers to set the coil and stimulation in place, but they are not aware if the treatment delivered is active or sham. This approach is further completed using robots being able to hold the coil in place on the target during the session guided by neuro-navigation. In the present study, most of the participants received rTMS without neuro-navigation and without robotic arms. Our pragmatic design allowed centres equipped with more technological tools to use them, as occurs in clinical practice. Importantly, we could not find significant differences in outcomes across the centres equipped or not with technologies such as neuro-navigation, card-based blinding procedures, or robots. This is in line with previous studies showing the absence of a higher placebo effect in patients receiving either the traditional or the card-based treatment allocation methodology^28^ and with studies demonstrating similar sham effects of different types of noninvasive cortical stimulation techniques (i.e. rTMS and transcranial direct current stimulation).^30^

The primary and secondary outcomes used reduction of at least 30% or 50% in scores from baseline as cutoff values to classify responders. However, for many pain assessment tools, the minimally clinically important difference is much lower than 30–50% changes. For example, for FIQ it is 14% change in total score,^31^ and for the HADS it is below 15%.^32^ The higher threshold to classify responders used must be contextualised when comparing the efficacy of nonpharmacological and pharmacological approaches for the management of fibromyalgia, and when running cost-effectiveness analyses in the future.

This study has some limitations. Despite the relatively long duration of treatment, fibromyalgia is a chronic condition, and it is clinically relevant to assess whether nonpharmacological treatments such as rTMS can be maintained for longer periods beyond 16 weeks. It is unknown if the positive effects of M1-rTMS would persist after the end of maintenance sessions. The assessment of symptom recrudescence and safety months after the end of therapy is a central issue that has not been comprehensively assessed in pharmacological and neuromodulation trials for fibromyalgia. This trial only included women with fibromyalgia. Although this might limit the generalisability of results to men with this condition, epidemiological studies show that women represent most people suffering from this condition (average 3:1 female-to-male ratio).^33^ Available data suggest that men present with more widespread pain and memory problems, higher fatigue, and with lower pressure pain thresholds,^34^ and therefore might be more inclined to be addressed with add-on nonpharmacological treatments such as neuromodulation. Although rTMS appeared safe in people with fibromyalgia, with a low rate of generally mild-to-moderate adverse events, larger studies with longer follow-up, and phase 4 real-life data are necessary to confirm safety findings. Finally, despite the use of dedicated and standardised forms to assess adverse events in each treatment session, symptoms that are not mentioned in the questionnaire might have occurred and not have been detected.

In conclusion, this is the first multicentre study which showed that M1-rTMS leads to a high probability of relevant pain relief in women with fibromyalgia up to 8 weeks, with a relatively high effect size and an apparently good safety profile. Although these data help include noninvasive brain stimulation approaches to pain relief among the treatment options for fibromyalgia, efforts are needed to better characterise the response profile and personalise this therapy to improve the success rate further.

## Authors’ contributions

Had full access to all of the data in the study and take responsibility for the integrity of the data and the accuracy of the data analysis: DCA; AFB, VAS

Concept and design of the study: VAS, DCA, NA, DB, AFB, KH, PC, ARB, GTK, NA, KMS

Acquisition, analysis, and interpretation of data: VAS, AMC, DCA

Drafting a significant portion of the manuscript or figures: VAS, AMC, DCA, AFB, NA, DB, ARB, GTK

Critically reviewed the manuscript and approved it in its final version: all authors

## Data availability

Deidentified individual participant data that support the findings reported in this article, and the study protocol and statistical analysis plan, are available at the discretion of the corresponding author upon reasonable request to researchers who provide a methodologically sound proposal up to 5 yr after publication. Proposals should be directed to dca@hst.aau.dk.

## Funding

LIM62 - Pain Center of the Department of Neurology at the University of São Paulo [to cover the costs of conducting the analyses and supporting the essential data management team for this trial]; CNPq [a scientific scholarship stipend to DCA, MJT, and AFB]; Teijin Pharma Limited [a joint research department, established with sponsorship to KH and NM, until March 31, 2021]; the Danish National Research Foundation (DNRF121) [to The Center for Neuroplasticity and Pain (CNAP)]; MagVenture [an 18-month partial PhD stipend to co-fund VAS under an investigator-initiated research project].

MagVenture had no role in the initiative to conduct the trial, its design, data collection, data analyses, drafting of the manuscript, revision of the manuscript, or role in the decision on where and when to publish it. MagVenture never had access to the data of the study before its publication. Using MagVenture machines or coils was never a prerequisite to have centres invited or joining the trial.

## Declaration of interests

NA is an editor of the *British Journal of Anaesthesia*. The other authors declare that they have no conflicts of interest.
