# Supplementary material for: Motor cortex repetitive transcranial magnetic stimulation in fibromyalgia: a multicentre randomised controlled trial
Source: Br J Anaesth. 2025 Mar 13;134(6):1756–64. doi: 10.1016/j.bja.2024.12.045 (PMC12106861; doi:10.1016/j.bja.2024.12.045)
Supplement: Multimedia component 1 [file mmc1.pdf]

Summary

Methods 1

Statistical analyses. 1

Figure S1. Study design... 2

Table S1. Stimulation parameters used in each center 3

Table S2. Baseline data across trial sites. 4

Table S3. Pain, functionality and global impression of change outcomes and on-going medication reduction during follow-up across study sites. 5

Table S4. Association between baseline characteristics and pain improvement with active repetitive Transcranial Magnetic Stimulation. 6

Table S5. Blinding assessment. 7

References. 8

Methods

Statistical analyses.

Before the start of the trial, we investigated different assumptions regarding the minimal difference to be found between the interventions (ranging from a 1.6 to 2.2 difference between arms) and different assumptions regarding standard deviations (both based on Mhalla *et al*[1]), a different number of interim analyses (from 0 to 2), different futility (when the trial is stopped and the interventions present only minor outcome differences) and success criteria (when the trial is stopped since one intervention presents a large difference to the other), different attrition rates (ranging from 20 to 80%, given that chronic pain patients are particularly prone to high dropout rates), as well as the use of different informative and non-informative priors for the difference between groups. When comparing different designs and scenarios, the optimal trial design was defined as two-arm comparisons of 40 participants included in each arm. To compensate for a dropout rate of up to 20%, we have increased the expected sample size to 50 patients per arm. During the recruitment phase of the study, the COVID-19 pandemic broke out, and the 32 patients had their treatments suspended as the study needed to be suspended and halted. After a meeting with the study´s steering board it was decided that these participants would not have their data analysed and were excluded from the study. Recruitment of new patients was resumed, and the study restarted after the pandemic. The online trial registry protocol was updated accordingly during the pandemic to reflect these choices.

Analyses were done according to intention-to-treat. Exploratory analysis started with visual exploration of all variables to evaluate the frequency, percentage, and near-zero variance for categorical variables (e.g., sex, race, and marital status), the distribution for numeric variables (e.g., NRS, GIC, BPI), and their respective missing value patterns[2]. Differences across study arms were assessed through standardized mean differences (SMD, where 0.2 corresponds to a small effect, 0.5 to a medium effect, and 0.8 to a large effect[54]. We considered a *p*-value of less than 0.05 as statistically significant. A Bayesian multilevel model was used to evaluate the effect of rTMS on pain outcomes considering the hierarchical structure of the data (i.e., multiple observations from the same participant over time), and previously used in this setting[3]. We interpreted the model results as a point estimate representing the magnitude of each treatment effect (i.e., regression coefficient) and an interval estimate for the precision of that estimate (median of the posterior distribution for each treatment, by using 95% credible intervals - CrI). Data imputation was made considering the multivariate imputation by chained equations (MICE) algorithm[4]. One hundred multiple imputations were performed by applying two imputation methods: logistic regression for binary variables and polytomous regression for categorical variables. Also, we used four chains of Markov Chain Monte Carlo (MCMC), with 1,000 warm-up iterations followed by 3,000 posterior sampling iterations per chain. We used weakly informative priors to derive the posterior distributions, and we set all models a priori and based on clinical assumptions using weakly informative priors. Secondary frequentist analyses were made for the primary outcome (Chi-square) and ANOVA/Student´s t tests for baseline data. All analyses were performed using the R statistical language with the Stan rstanarm package[5].

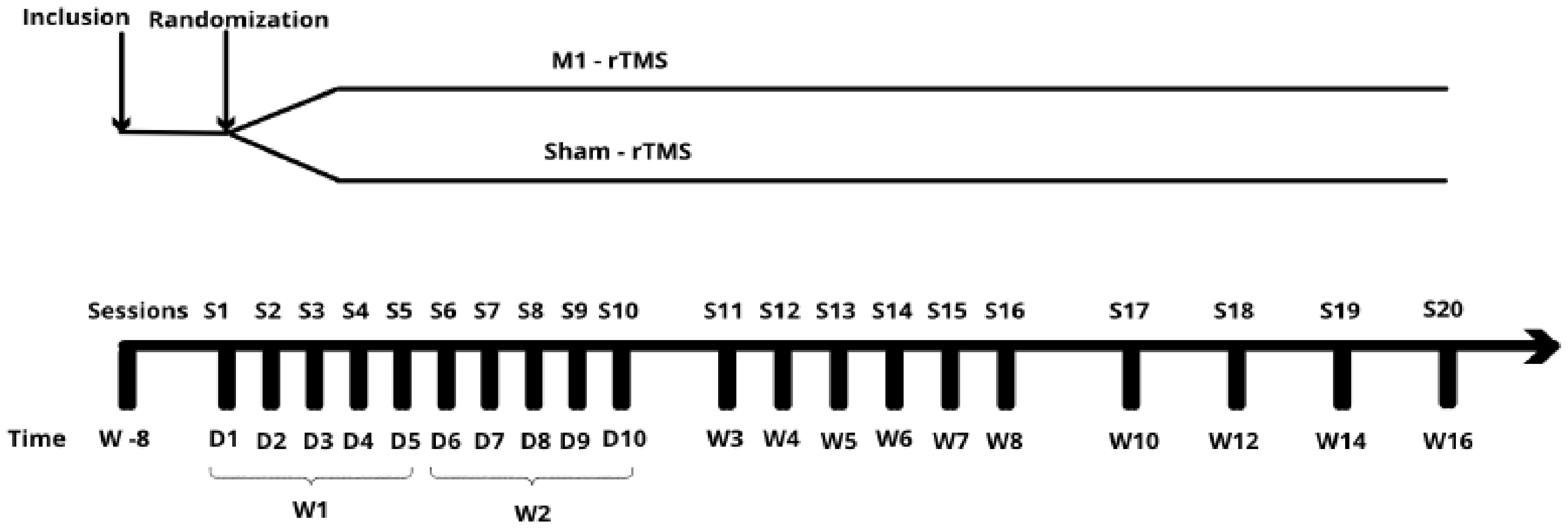

Figure S1. Study design.

This figure summarizes the general design of this trial. After baseline assessment, participants were randomized into two parallel arms to receive either active repetitive transcranial magnetic stimulation targeting the primary motor cortex (M1-rTMS) or sham treatment (Sham-rTMS). Treatments were delivered for 10 days (D1 to D10) throughout 2 consecutive weeks (W1 and W2); followed by once weekly sessions from W3 to W8; and then by sessions every other week from W10 to W16. Clinical assessments were performed at baseline, W8 and W16. These included a structured sociodemographic questionnaire (only at baseline); Brief Pain Inventory - short form; Fibromyalgia Impact Questionnaire; Global Impression of Change (clinician and participant versions). The Brief Pain Inventory - short form and the Hospital Anxiety and Depression Scale were applied at baseline and W8; side-effects were inquired at every treatment session, and blinding was assessed on the last day of the study (W16). M1 – primary motor cortex; rTMS – repetitive Trascranial Magnetic Stimulation; * - treatment sessions (*); D – day; W - week.

Table S1. Stimulation parameters used in each center

Study Centre	rTMS device	Active Coil	Sham Coil	Use of neuronavigation system	Use of robot arm
Brazil - Neurology Department	MagPro X100 (MagVenture, Denmark)	Figure-of-eight coil, (Cool-B-65 coil MagVenture, Denmark)	Figure-of-eight coil, (MC-P-B70, MagVenture, Denmark)	No	No
Brazil - Physiotherapy Department	MagPro X100 (MagVenture, Denmark)	Figure-of-eight coil, (Cool-B-65 coil MagVenture, Denmark)	Figure-of-eight coil, (MC-P-B70, MagVenture, Denmark)	No	No
Brazil - Federal University of Pernambuco	MagPro R20 (MagVenture, Denmark)	Figure-of-eight coil, (MCF-B-70 coil MagVenture, Denmark)	Figure-of-eight coil, (B-70 coil MagVenture, Denmark)	No	No
State University of Western Paraná	MagPro R20 (MagVenture, Denmark)	Figure-of-eight coil, (MCF-B-70 coil MagVenture, Denmark)	Figure-of-eight coil, (B-70 coil MagVenture, Denmark)	No	No
Japan	MagPro X100 (MagVenture, Denmark)	Figure-of-eight coil (MC-B70, MagVenture, Denmark)	Figure-of-eight coil (MC-P-B70, MagVenture, Denmark)	Yes (Brainsight; Rogue Research, Inc., Montreal, QC, Canada)	No
France	MagPro X100 (MagVenture, Denmark)	Cool-B65 A/P figure-of-8 coil (MagVenture, Denmark)	Cool-B65 A/P figure-of-8 coil (MagVenture, Denmark)	Yes (Syneika ONE, Syneika; or visor2TM, ANT Neuro)	Yes (Axilum Robotics)

A/P: Active/Placebo. *rTMS – repetitive Transcranial Magnetic Stimulation.*

Table S2. Baseline data across trial sites.

	All study centres (n = 101)	Brazil - Neurology Department (n = 48)	France (n = 12)	Brazil - Federal University of Pernambuco (n = 7)	Japan (n = 10)	Brazil – Physiotherapy Department (n = 22)	Brazil - State University of Western Paraná (n = 2)	p
Average Pain Intensity NRS	7.82 (±1.23)	7.88 (±1.04)	7.33 (±1.15)	7.71 (±1.5)	6.9 (±1.73)	8.45 (±1.06)	7.5 (±0.707)	0.167
BPI Average Pain	7.21 (±1.51)	7.17 (±1.48)	6.75 (±1.42)	8 (±1.53)	6.6 (±1.78)	7.55 (±1.47)	7.5 (±0.707)	0.512
BPI Pain relief with medication during the last 24h	28.9 (±24.9)	26.2 (±20.9)	25.8 (±24.3) [	41.4 (±24.1)	16 (±19)	36.4 (±32.4)	50 (±28.3)	0.312
BPI Pain Interference Score	7.96 (±2.12)	8.54 (±1.67)	5.4 (±2.16)	8.22 (±1.45)	6.51 (±2.71)	8.94 (±1.13)	4.86 (±2.02)	0.011
HADS Anxiety	9.23 (±4.41)	8.71 (±4.44)	10.6 (±4.03)	12.6 (±3.51)	9.1 (±4.82)	8.68 (±4.54)	8.5 (±0.707)	0.167
HADS Depression	8.98 (±4.25)	9.12 (±4.44)	8.25 (±4.33)	12 (±2.58)	7.6 (±4.33)	8.82 (±4.12)	8 (±4.24)	0.248
FIQ	68.5 (±12.8)	68.6 (±13.1)	60.2 (±11)	77.8 (±10.5)	61.8 (±10.4)	72.6 (±11.8)	72.7 (±13.2)	0.073
Medication use at baseline [A]								
Antidepressants	39 (38.6)	18 (37.5)	8 (66.7)	4 (57.1)	2 (20)	7 (31.8)	0 (0)	0.147
Gabapentinoids	25 (24.8)	15 (31.2)	0 (0)	2 (28.6)	2 (20)	6 (27.3)	0 (0)	0.312
Opiates	19 (18.8)	11 (22.9)	2 (16.7)	0 (0)	3 (30)	3 (13.6)	0 (0)	0.570

Data are presented as mean (± standard-deviation), unless otherwise stated.
[A]Data presented as n (%).
NRS – verbal numerical rating scale; BPI – Brief Pain Inventory; HADS – Hospital Anxiety and Depression Scale; FIQ – Fibromyalgia Impact Questionnaire

Table S3. Pain, functionality and global impression of change outcomes and on-going medication reduction during follow-up across study sites.

	All study centres (n = 101)	Brazil - Neurology Department (n = 48)	France (n = 12)	Brazil - Federal University of Pernambuco (n = 7)	Japan (n = 10)	Brazil – Physiotherapy Department (n = 22)	Brazil - State University of Western Paraná (n = 2)	p
Week 8								
Average Pain Intensity NRS	5.16 (±2.23)	5.12 (±2.09)	4.58 (±2.57)	6.71 (±1.38)	5.1 (±2.23)	4.91 (±2.43)	7 (±2.83)	0.271
BPI pain severity	5.4 (±1.91)	5.58 (±1.67)	4.31 (±2.5)	7.01 (±0.95)	4.95 (±2)	5.28 (±1.94)	5.62 (±2.65)	0.072
FIQ	50.2 (±17.8)	49.3 (±19.5)	41.8 (±14.5)	64 (±12.1)	52.2 (±14.6)	50.9 (±16.6)	56.1 (±28)	0.163
HADS Anxiety	7.44 (±4.48)	6.23 (±4.46)	8.75 (±5.17)	11.4 (±3.51)	7 (±3.02)	8.27 (±4.37)	7.5 (±2.12)	0.129
HADS Depression	7.72 (±4.51)	7 (±4.57)	7.5 (±4.58)	12 (±3.21)	7.4 (±4.4)	8.59 (±4.14)	3.5 (±4.95)	0.120
Improvement in patient GIC	2.5 (±1.15)	2.31 (±1.01)	3.17 (±1.47)	2.29 (±1.11)	3.3 (±0.949)	2.27 (±1.08)	2.5 (±2.12)	0.191
Improvement in clinician GIC	2.9 (±1.05)	2.94 (±0.976)	3.08 (±1.16)	2.86 (±1.07)	3.2 (±0.919)	2.64 (±1.18)	2.5 (±-2.12)	0.842
Week 16								
Average Pain Intensity NRS	5.59 (±2.33)	5.75 (±2.51)	4.92 (±0.669)	6.57 (±1.51)	5.8 (±2.1)	5.23 (±2.88)	5.5 (±0.707)	0.207
FIQ	53.4 (±15.6)	51.5 (±17.2)	51.3 (±2.57)	63 (±13.3)	56.9 (±15.1)	52.5 (±15.7)	67.6 (±22)	0.396
Improvement in clinician GIC	2.74 (±1.07)	2.92 (±0.986)	2.33 (±0.985)	2.86 (±1.07)	3.1 (±1.2)	2.41 (±1.14)	2.5 (±2.12)	0.477
Improvement in patient GIC	2.38 (±1.08)	2.29 (±0.967)	2.17 (±1.03)	2.57 (±0.976)	3.2 (±1.14)	2.23 (±1.23)	2.5 (±2.12)	0.456

Data are presented as mean (± standard-deviation), unless otherwise stated.
[A]Data presented as n (%). NRS – verbal numerical rating scale; BPI – Brief Pain Inventory; FIQ – Fibromyalgia Impact Questionnaire; HADS – Hospital Anxiety and Depression Scale; GIC – Global Impression of Change
* $p<0.05$

.

Table S4. Association between baseline characteristics and pain improvement with active repetitive Transcranial Magnetic Stimulation.

Variable	Total (n = 52)	Pain improved by at least 50% (n = 21)	Pain not improved by at least 50% (n = 31)	p
Age (years)[A]	49 (±12.3)	51.6 (±10)	47.3 (±13.4)	0.186
Race				
White	25 (48.1)	12 (57.1)	13 (41.9)	
Black	12 (23.1)	5 (23.8)	7 (22.6)	0.589
Asian	5 (9.6)	1 (4.8)	4 (12.9)	
Other	10 (19.2)	3 (14.3)	7 (22.6)	
Education				
Elementary	7 (13.5)	2 (9.5)	5 (16.1)	
Middle	11 (21.2)	6 (28.6)	5 (16.1)	0.660
High	14 (26.9)	6 (28.6)	8 (25.8)	
Undergraduate or more	20 (38.5)	7 (33.3)	13 (41.9)	
Medication use				
Anticonvulsants	8 (15.4)	2 (9.5)	6 (19.4)	0.567
Antidepressants	18 (34.6)	6 (28.6)	12 (38.7)	0.648
Gabapentinoids	14 (26.9)	4 (19.0)	10 (32.3)	0.462
Opiates	11 (21.2)	1 (4.8)	10 (32.3)	0.042*
Neuroleptics	3 (5.8)	1 (4.8)	2 (6.4)	1.000
Metamizole	10 (19.2)	2 (9.5)	8 (25.8)	0.270
Cognitive Therapy	18 (34.6)	8 (38.1)	10 (32.3)	0.958
Physical Therapy	28 (53.8)	11 (52.4)	17 (54.8)	0.987

Data presented as n (%), unless otherwise stated.
[A]Data are presented as mean (± standard-deviation).
* $p<0.05$

Table S5. Blinding assessment.

	rTMS (n = 52)	sham (n = 49)	p
Experience of pain during procedure (0-10)	1 (±2.55)	1.14 (±2.53)	0.814
Affirmed that could guess which treatment received	22 (42.3)	19 (38.8)	1.000
Type of procedure believed to have received			
rTMS	26 (70.3)	25 (71.4)	1.000
Sham	11 (29.7)	10 (28.6)	
Reported what would like to undergo the procedure again	38 (73.1)	34 (69.4)	1.000

Data presented as n (%), unless otherwise stated.
[A]Data are presented as mean (± standard-deviation).
rTMS – repetitive Transcranial Magnetic Stimulation

References.

1 Mhalla A, Baudic S, De Andrade DC, *et al.* Long-term maintenance of the analgesic effects of transcranial magnetic stimulation in fibromyalgia. *Pain* 2011; **152**: 1478–85.

2 Kuhn M, Johnson K. Applied Predictive Modeling, 1st edn. New York: Springer New York, 2013.

3 Hamani C, Fonoff ET, Parravano DC, *et al.* Motor cortex stimulation for chronic neuropathic pain: results of a double-blind randomized study. *Brain* 2021; **144**: 2994–3004.

4 van Buuren S. Flexible Imputation of Missing Data, 1st edn. New York: Chapman and Hall/CRC, 2012 DOI:10.1201/b11826.

5 Team RC, Team RC, Others. A language and environment for statistical computing. R Foundation for Statistical Computing, Vienna, Austria. 2013.
